# Efficacy of Duloxetine for Postspine Surgery Pain: A Systematic Review and Meta‐Analysis

**DOI:** 10.1002/brb3.70217

**Published:** 2024-12-31

**Authors:** Abdulsalam Mohammed Aleid, Faisal Alshehri, Naif Alasiri, Fatimah Alhomoud, Shouq Alsaegh, Mohammed Alrasheed, Salem Aljaddua, Ali Alasiri, Asma Boukhari, Abdulmonem Ali Alhussain, Bipin Chaurasia, Saud Nayef Aldanyowi

**Affiliations:** ^1^ Department of Surgery, Medical College King Faisal University Hofuf, Ahsa Saudi Arabia; ^2^ Department of surgery King Khalid University Abha Saudi Arabia; ^3^ Department of surgery Imam Abdulrahman Bin Faisal University Dammam Saudi Arabia; ^4^ Department of surgery Qassim University Unaizah Saudi Arabia; ^5^ Department of surgery King Saud Bin Abdulaziz University for Health Sciences Riyadh Saudi Arabia; ^6^ Department of surgery Jouf University Aljouf Saudi Arabia; ^7^ Department of surgery Rijal Almaa General Hospital Rijal Almaa Saudi Arabia; ^8^ Department of Neurosurgery King Fahad Hospital Hofuf, Ahsa Saudi Arabia; ^9^ Department of Neurosurgery Neurosurgery Clinic Birgunj Nepal

**Keywords:** duloxetine, pain, spine, surgery

## Abstract

**Background:**

Duloxetine, a serotonin and norepinephrine reuptake inhibitor (SNRI), is used to treat various health conditions, including major depressive disorder, generalized anxiety disorder, fibromyalgia, and off‐label for chemotherapy‐induced pain. We conducted this systematic review and meta‐analysis aiming to test the current evidence regarding effectiveness and safety of duloxetine for postspine surgeries pain.

**Methods:**

We searched the Cochrane Central Register of Controlled Trials, PubMed, Scopus and Web of science databases for relevant articles up to March 2024. The following search terms were Used in combination using the Boolean operators ((Duloxetine Hydrochloride) AND ((Pain, Postoperative) OR (Postoperative Period) OR (Postoperative Cognitive Complications) OR (Delayed Emergence from Anesthesia) OR (Postoperative Care) OR (spine surgery)) without time constrain for the search. Meta‐analysis was performed using Review Manager (RevMan version 5.4) on the extracted outcome data that present in at least 3 of the included studies. Mean difference (MD) was used as the effect size for continuous outcomes with a 95% confidence interval (CI) or standardized mean difference (SMD) in case of different outcome reporting scales.

**Results:**

Pooled analysis showed that duloxetine significantly reduces pain intensity after 24 h from the operation compared to placebo (SMD = −1.11, 95% CI [−2.16 to −0.07], *p* = 0.04) with no significant difference in pain after 2 and 48 h. Meta‐analysis revealed that duloxetine shows a significant reduction in the amount of analgesic consumption after 24 h postoperative; (MD = −3.33, 95% CI [−5.53 to −1.13], *p* = 0.003). The analysis did not show any statistically significant difference between duloxetine and placebo in patients experiencing nausea or vomiting (RR = 1.37, 95% CI [0.62 to 3.00]

**Conclusion:**

The findings of this study suggest that duloxetine may be effective in reducing pain 24 h after spine surgery. Furthermore, there is a promising effect of duloxetine in treating chronic postoperative pain. However, it is important to acknowledge that further research is warranted to thoroughly evaluate the efficacy and safety of duloxetine for relieving chronic postoperative pain.

## Graphical abstract

Image showing 465 article has been published for efficacy of duloxetine in reducing postoperative spine pain. Many paper shows duloxetine significantly reduces pain intensity after 24 h from the operation compared to placebo.

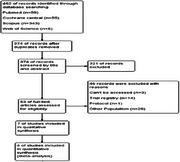

## 1 Introduction

Postsurgical pain is a normal body response to surgical intervention, triggered by tissue trauma or direct nerve injury (Lovich‐Sapola, Smith, and Brandt 2015). Despite advancements in medical care, pain relief after surgery continues to be a major medical challenge. Not only it constitutes a contributing factor to delayed recovery and discharge after surgery, but it also increases the risk of wound infection and respiratory/cardiovascular complications. Moreover, it limits patients’ ability to participate in rehabilitation programs (Rawal 2016). The scale of this challenge is underscored by findings from both a recent meta‐analysis and the US National Institutes of Health. The recent meta‐analysis provided estimates of pooled prevalence rates of moderate‐to‐severe postoperative pain after hospital discharge from 31% to 58% (Park et al. 2023). Concurrently, The NIH report reveals that a substantial majority of patients, exceeding 80%, encounter postoperative pain, yet less than half receive adequate relief (Institute of Medicine (US) Committee on Advancing Pain Research, Care, and Education 2011).

Spinal surgeries often involve extensive manipulation of subcutaneous tissues, bones, and ligaments leading to extensive postoperative pain. The pain is severe and typically lasts for 3 days (Nielsen et al. 2014). A prospective observational study demonstrated that more than half of adults undergoing back surgery continue to report moderate pain 6 months after their operation (Laufenberg‐Feldmann et al. 2016). Therefore, providing adequate pain relief is crucial in the postoperative care of these patients. However, Pain management after spine surgery is challenging as patients undergoing spine surgeries often present with preexisting chronic pain and dependence of analgesic and/or narcotics. The preexisting pain along with long‐term consumption of analgesics and/or opioids alters pain perception in these patients thereby complicating pain management strategies (Bhaskar and Bajwa 2013).

Traditionally, opioids have been widely prescribed postoperatively for pain control, despite the increasing recognition of the benefits of multimodal pain management plans. Opioid therapy can result in several adverse effects including nausea, vomiting, constipation, ileus, urinary retention, sedation, and respiratory depression (Devin and McGirt 2015) and most importantly opioid‐related adverse effects including persistent postoperative opioid use, opioid misuse, and diversion of surplus opioid prescriptions (Prabhakar et al. 2022). A retrospective review based on more than 300,000 patients across 380 US hospitals showed that about 95% of surgical patients were treated with opioids and 12.2% of them showed opioid related adverse events (Oderda et al. 2013). As discussed before, spine surgeries often lead to intense postoperative pain and high postoperative opioid use, especially for patients with preexisting chronic pain or opioid use prior to surgery (Nielsen et al. 2014). In turn, these patients are at greater risk of adverse surgical outcomes including increased length of hospital stay, increased surgical site infections and increased reoperations (Samuel et al. 2021). Approximately 9% of patients continue to use opioids 1 year after spine surgery (Kowalski et al. 2023). Hence, it is essential to transition toward utilization of nonopioid analgesics.

Duloxetine, a serotonin and norepinephrine reuptake inhibitor (SNRI), is used to treat various health conditions, including major depressive disorder, generalized anxiety disorder, fibromyalgia, and off‐label for chemotherapy‐induced pain. Recently, it has been widely used in the surgical field for pain management. The rationale behind its use in postoperative pain management lies in its ability to enhance the activity of noradrenergic and serotonergic neurons in the descending spinal pathway, thereby inhibiting dorsal horn neuron activity and suppressing excessive pain signals reaching the brain (Kiso et al. 2018). Additionally, SNRIs like duloxetine demonstrate potent anti‐platelet, endothelial protection properties and regulate the production of interleukin and interferon, contributing to wound healing (Fields, Heinricher, and Mason 1991; Woolf 2004).

Previous research has tested the effect of duloxetine and other SNRIs in wide field of surgeries. Zhou and colleagues in their meta‐analysis have reviewed CTs testing Duloxetine in total knee or total hip arthroplasty (TKA or THA) and concluded that duloxetine could lower opioid consumption and relieve postoperative pain (Zhou et al. 2023). However, Nair et al. highlighted that although duloxetine has statistically significant pooled treatment effect in pain posthysterectomy, the evidence was not conclusive to advocate routine duloxetine premedication before hysterectomy (Nair et al. 2023). There is no current systematic review and meta‐analysis evaluating duloxetine as a potential treatment for postspine surgery pain.

We conducted this systematic review and meta‐analysis aiming to test the current evidence regarding effectiveness and safety of duloxetine for postspine surgeries pain.

## 2 Materials and Methods

This study was conducted according to the Preferred Reporting Items for Systematic Reviews and Meta‐Analyses (PRISMA) guidelines (Page et al. 2021) and the recommendations of the Cochrane Handbook for Systematic Reviews of Interventions (Higgins et al. 2023). This study was registered with PROSPERO (CRD42024536148)

### 2.1 Database Searching

We searched the Cochrane Central Register of Controlled Trials, PubMed, Scopus, and Web of science databases for relevant articles up to March 2024. The following search terms were used in combination using the Boolean operators ((Duloxetine Hydrochloride) AND ((Pain, Postoperative) OR (Postoperative Period) OR (Postoperative Cognitive Complications) OR (Delayed Emergence from Anesthesia) OR (Postoperative Care) OR (spine surgery)) without time constrain for the search

### 2.2 Inclusion and Exclusion Criteria

#### 2.2.1 Inclusion Criteria

For quantitative assessment, we included all RCTs comparing duloxetine used at any dose and regimen against placebo administered perioperative in patients aged 18 years or older undergoing any spinal cord surgeries including cervical, lumbar, or other spinal cord surgeries. RCTs measuring postoperative pain and/or analgesia utilization 24 h postoperatively are included in our study.

#### 2.2.2 Exclusion Criteria

Articles with designs other than RCTs such as reviews, letters to editors, abstracts, opinions, and nonhuman studies were excluded from meta‐analysis. Trials used drugs other than duloxetine, additional interventional drugs, other comparators instead of placebo or not published in English were also excluded.

### 2.3 Study Section and Data Extraction

Search results were exported to Endnote software version 20 (The EndNote Team 2013) where duplicates were screened and removed before using Rayyan software (Ouzzani et al. 2016) for initial review of titles and abstracts of all search results independently by two authors to assess their eligibility for the study. Any disagreement between the two reviewers was solved by discussion. The final decision was made by a third reviewer if the disagreement continued. Full‐text screening was conducted to the eligible articles which met the inclusion criteria. References of final included studies were retrieved to avoid omitting potential additional studies not included in the initial search. The data of final records including year of publication, target population, baseline characteristics, study locations and outcomes Were extracted manually from the articles into a Google Sheet by two independent reviewers.

### 2.4 Efficacy Outcomes

The primary outcome of this study was postoperative pain at 2, 24, and 48 h after operation. Secondary outcomes were analgesics consumption or requirements within 24 h after surgeries and time and time to rescue analgesia requirement in the first 24 h postoperatively.

### 2.5 Safety Outcomes

Assessed adverse events focused on patients experiencing gastrointestinal symptoms (nausea and vomiting), Somnolence, itching and purities after surgeries

### 2.6 Risk of Bias Assessment

The risk of bias of included clinical trials was conducted according to Cochrane Risk of Bias tool (ROB1) of interventional studies stated in their book, which encompasses selection bias and allocation concealment, blinding of patients and personnel, Blinding of outcome assessors, missing outcomes data, selective reporting of outcomes and other sources of bias if present. Each domain was rated as a high, low, or unclear risk by two authors independently. Nonrandomized observational studies was evaluated using Newcastle–Ottawa scale (Wells et al. 2013) which encompasses assessment of Selection, Comparability and Outcome. Disagreements were resolved with collegial discussion.

### 2.7 Statistical Analysis

Meta‐analysis was performed using Review Manager (RevMan version 5.4) (RevMan) on the extracted outcome data that present in at least 3 of the included studies. Mean difference (MD) was used as the effect size for continuous outcomes with a 95% confidence interval (CI) or standardized mean difference (SMD) in case of different outcome reporting scales. For dichotomous outcome data, the frequency of events and total number of patients were pooled as risk ratio (RR) with 95% CI. To account for in‐between studies heterogeneity and obtain a more conservative effect estimate, we adopted a random effect model rather than a fixed effect model. Heterogeneity was assessed by the chi‐square test with a probability value of *p* ≤ 0.1 and an *I* ^2^ value > 40%. We concerned more with *I* ^2^ value as *I* ^2^ test acts better than chi‐square test when the number of included studies is small. To solve heterogeneity, sensitivity analysis was performed.

## 3 Results

### 3.1 Study Selection

**FIGURE 1:**
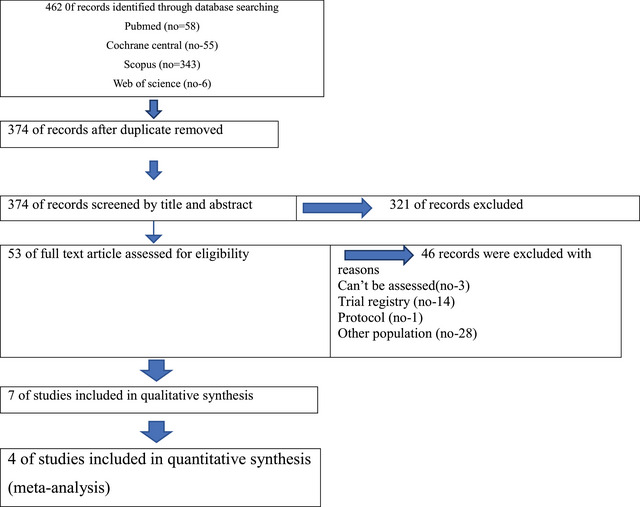
PRISMA flow diagram.

Searching the literature systematically shows/yields 462 potentially relevant records from various databases including PubMed, Cochrane central, Scopus and Web of Science. Endnote was utilized for removing duplicates which appear to be 88 records. Titles and abstracts screening was conducted to 374 records yielding 53 articles which met the eligibility criteria for our research question. The final step taken was the full text assessment and 46 records were excluded for reasons. Therefore, seven studies (Attia and Mansour 2017; Bedin et al. 2017; Altiparmak, Güzel, and Gümüş Demirbilek 2018; Govil et al. 2020; Hiroki et al. 2022; Tsuji et al. 2021; Liu et al. 2023) were included in the qualitative synthesis while four of them were included in the meta‐analysis. The process of searching as well as the number of included and excluded studies are shown in Figure 1.

### 3.2 Characteristics of the Studies

All studies are published between 2017 and 2023. Five of the included (Attia and Mansour 2017; Bedin et al. 2017; Altiparmak, Güzel, and Gümüş Demirbilek 2018; Govil et al. 2020; Hiroki et al. 2022) studies are RCTs and evaluate the effectiveness of preoperative duloxetine in reducing postoperative pain after spine surgeries, while the remaining two (Tsuji et al. 2021; Liu et al. 2023) are mixed cohort evaluating the effect of postoperative duloxetine. The four included studies in the qualitative synthesis compared duloxetine against placebo are (Attia and Mansour 2017; Bedin et al. 2017; Altiparmak, Güzel, and Gümüş Demirbilek 2018; Govil et al. 2020). Tsuji et al. (2021) design was a single cohort so there was no control group. Hiroki et al. (2022) compared against diazepam while Liu et al. (2023) compared against different active drugs. Interventional groups were treated with single dose of duloxetine 60 mg orally. The overall population was 273 participants who underwent various spine surgeries. Summary of included studies are shown in Table 1 and baseline characteristics of patients in Table 2.

**TABLE 1:** Summary of included studies.

Study ID	Study design	Country	Surgery	Interventions	Control	Sample size		Main results
						Intervention	Control	
Altıparmak 2018	RCT	Turkey	Repair of lumbar disc herniation	**‐ Duloxetine**: 1 h before surgery → duloxetine 60 mg orally 12th h postoperative → identical placebo 24th h postoperative → duloxetine 60 mg orally **‐ Pregabalin**: 1 h before surgery, 12th and 24th h postoperative → pregabalin 75 mg orally	Placebo	Duloxetine (*n* = 31) Pregabalin (*n* = 30)	Placebo (*n* = 33)	The preoperative administration of duloxetine 60 mg may serve as a beneficial alternative to pregabalin 75 mg, as both exhibit comparable analgesic effects on postoperative pain, but duloxetine is associated with a reduced incidence of drug‐related adverse effects on cognitive function.
Attia 2017	RCT	Egypt	Single level lumbar spinal disc prolapse	**‐ Etoricoxib**: received placebo capsule + two etoricoxib tablet 60 mg (1 h before surgery and repeated after 24 h) **‐ Duloxetine**: received duloxetine capsule 60 mg + two placebo tablet (1 h before surgery and repeated after 24 h) **‐ Duloxetine + Etoricoxib**: received duloxetine 60 mg capsules + two etoricoxib tablets 90 mg (1 h before surgery and repeated after 24 h)	Placebo	Duloxetine (*n* = 30) Etoricoxib (*n* = 30) D+E (*n* = 30)	Placebo (*n* = 30)	The perioperative use of etoricoxib and duloxetine enhanced analgesia and diminished opioid usage without notable adverse effects.
Bedin 2017	RCT	Brazil	One segment lumbar spinal fusion	**‐ Duloxetine**: 60 mg 1 h before surgery and was administered again after 24 h	Placebo	Duloxetine (*n* = 28)	Placebo (*n* = 29)	Duloxetine proved helpful as an adjuvant for postoperative analgesia and diminished opioid intake.
Govil 2020	RCT	India	Lumbar canal stenosis surgery	**‐ Duloxetine**: oral duloxetine 30 mg once a day (OD) for 2 days before surgery. The dose was incremented to 60 mg OD from the day of surgery (postoperative day [POD] 0) to the second postoperative day (POD 2), again tapered to 30 mg OD for the next 2 days, i.e., until POD 5 (total duration 7 days)	Placebo	Duloxetine (*n* = 46)	Placebo (*n* = 46)	Duloxetine alleviates postoperative pain following lumbar canal stenosis surgery without elevating unwanted effects.
Hiroki 2022	RCT	Japan	Posterior lumbar interbody fusion surgery	**‐Duloxetine**: 40 mg 2 h before entering the operating room	diazepam 4 mg	Duloxetine (*n* = 22)	Diazepam (*n* = 18)	A solitary preoperative dose of 40 mg of duloxetine did not enhance postoperative pain following posterior lumbar interbody fusion surgery, but helped alleviate lower limb numbness. Duloxetine may alleviate neuropathic pain‐like sensations following posterior lumbar interbody fusion surgery.
Liu 2023	Retrospective observational study	China	Posterior cervical decompression surgery	**‐ Duloxetine**: 30 mg orally in the first week and 60 mg orally from the second week	Nondrug therapy group (health education, functional exercise and cervical collar wearing)	Duloxetine (*n* = 35)	Nondrug therapy (*n* = 28)	Oral duloxetine yields superior short‐term results compared to traditional nonpharmacological therapy in patients exhibiting axial symptoms post‐posterior decompression surgery of the cervical spine.
Tsuji 2021	Retrospective observational study	Japan	Spine or spinal cord surgery	**‐ Duloxetine**: initiated at 20 mg/day and gradually increased to a maximum of 60 mg/day, according to each patient's degree of adverse effects	No comparator	Duloxetine (*n* = 24) Effective group with NRS reduction ≥ 2 (*n* = 19) Non effective group with NRS reduction < 2 (*n* = 5)		This study evaluates the efficacy of duloxetine for persistent neuropathic problems following surgery. Duloxetine shown efficacy in alleviating postsurgical chronic pain (100%) and numbness (78.3%) in select patients; however, additional research is required to ascertain its appropriate application.

**TABLE 2:** Baseline characteristics of patients in included studies.

**Study ID**	**Study arms**	**Sample size**	**Age** **mean ± SD**	**Sex, females** ***n* (%)**	**BMI** **mean ± SD**	**ASA class I** ***n* (%)**	**ASA class II** ***n* (%)**	**Duration of Surgery in minutes** **mean ± SD**	**Preoperative pain score** **mean ± SD**
Altıparmak 2018	Duloxetine	31	53 ± 11	20 (64.5%)	28 ± 3	13 (40.6 %)	18 (29 %)	90 ± 9	5 ± 1
	Placebo	33	54 ± 11	21 (63.6 %)	28 ± 3	10 (31.3 %)	23 (37.1 %)	89 ± 9	5 ± 1
Attia 2017	Duloxetine	30	48.36 ± 9.8	12 (40%)	29.5 ± 11.1^†^	17 (56.67 %)	9 (30 %)	113.2 ± 13.7	NR
	Placebo	30	46.5 ± 8.74	15 (50%)	29 ± 11.1^†^	18 (60 %)	7 (23.3 %)	109.9 ± 10.8	NR
Bedin 2017	Duloxetine	28	48 ± 12	15 (53.5%)	29 ± 5	12 (42.85 %)	16 (57.15 %)	161 ± 55	1.51 ± 2.07
	Placebo	29	48 ± 14	16 (55.17%)	27 ± 5	6 (20.69 %)	21 (72.4 %)	166 ± 65	1.08 ± 1.08
Govil 2020	Duloxetine	46	41.40 ± 14.6	23 (50%)	25.90 ± 2.3	20 (43.48 %)	26 (56.52%)	113.4 ± 19.8	NR
	Placebo	46	61 ± 15.1	24 (52.17%)	26.60 ± 2.2	18 (39.13 %)	28 (60.87 %)	108 ± 22.2	NR
Hiroki 2022	Duloxetine	22	61.8 ± 15.2	8 (36.4%)	24.8 ± 2.5	7 (31.8 %)	15 (68.2 %)	107 ± 28.8	43 ± 29.6
	Diazepam	18	67.2 ± 10.03	8 (44.4%)	24.8 ± 3.7	7 (38.9 %)	11 (61.1 %)	120.8 ± 54.3	55.4 ± 23.8
Liu 2023	Duloxetine	35	66.6 ± 5.8	7 (20%)	26,06 ± 1.41	NR	NR	NR	6.61 ± 1.16
	Nondrug therapy	28	65.7 ± 5.26	7 (25%)	25,67 ± 2.32	NR	NR	NR	6.31 ± 1.4
Tsuji 2021	Duloxetine E	19	66.9 ± 11.2	7 (36.84%)	24.4 ± 4	NR	NR	NR	NR
	Duloxetine N	5	68.4 ± 11.5	2 (40%)	22.6 ± 2.6	NR	NR	NR	NR

### 3.3 Risk of Bias and Quality Assessment

**FIGURE 2:**
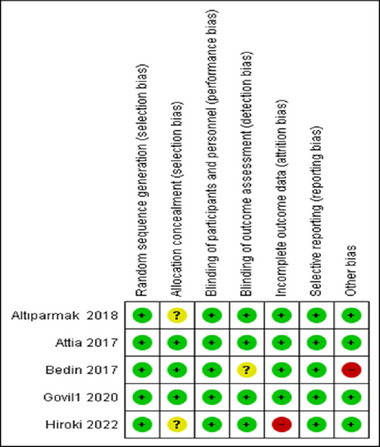
Summary of risk of bias of the included randomized controlled trials using ROB 1.

**FIGURE 3:**
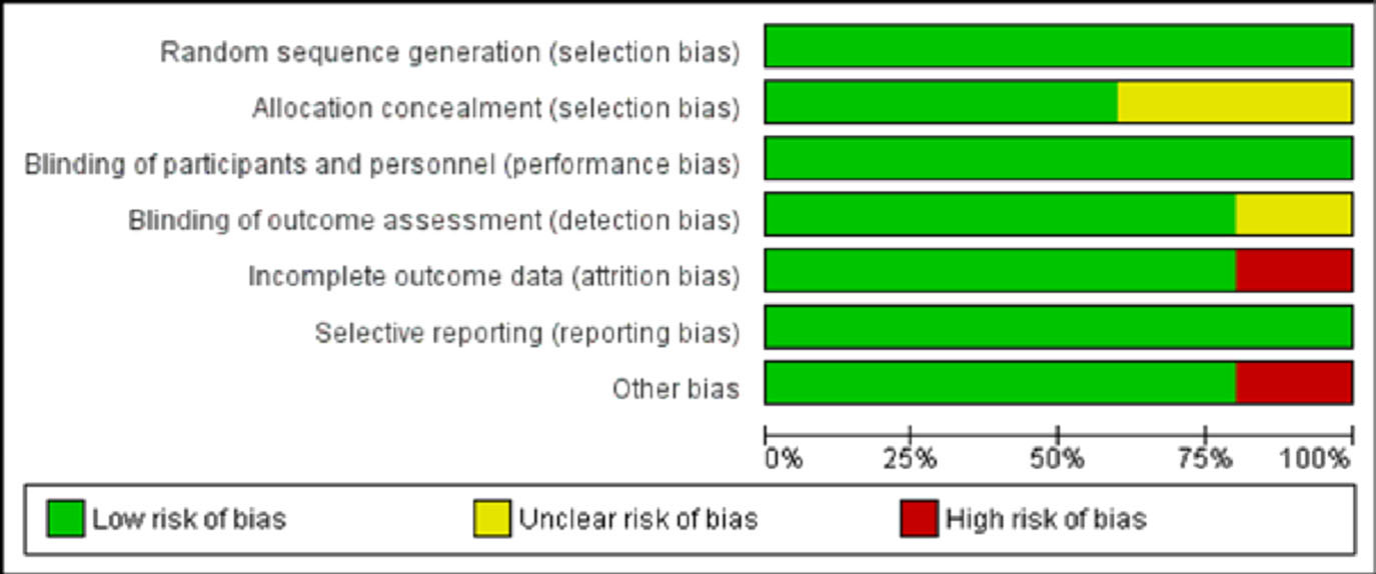
Risk of bias graph of the included randomized controlled trials using ROB and showing different domains.

Randomized controlled trials (Attia and Mansour 2017; Bedin et al. 2017; Altiparmak, Güzel, and Gümüş Demirbilek 2018; Govil et al. 2020; Hiroki et al. 2022) were assessed by ROB 1 while cohort studies (Tsuji et al. 2021; Liu, Yang, and Jing 2023) by new castle Ottawa scale (NOS). Bedin et al. (2017) and Hiroki et al. (2022) are considered to have high risk of bias, while there are some concerns about risk of bias in Altiparmak, Güzel, and Gümüş Demirbilek (2018) due to unclear information about allocation concealment. Attia and Mansour (2017) and Govil et al. (2020) were considered of high quality as shown in Figures 2 and 3. Tsuji et al. (2021) had a quality score of 9 stars while Liu et al. (2023) had 8 in NOS as shown in Table 3.

**TABLE 3:** Risk of bias assessment of nonrandomized studies using Newcastle–Ottawa scale.

Study ID	NOS (cohort) (coded)								
	Selection				Comparability	Outcome			Overall score
	Representativeness of the exposed cohort	Selection of the nonexposed Cohort	Ascertainment of the exposure (risk factor)	Demonstration that outcome of interest was not present at start of study	Comparability of cohorts on the basis of the design or analysis	Assessment of outcome	Was follow‐up long enough for outcomes to occur	Adequacy of follow up of cohorts	
**Tsuji 2021**	*	*	*	*	**	*	*	*	9 stars
**Liu 2023**	*	*	*	*	**	**0**	*	*	8 stars

*‐no risk

**‐medium risk

***‐high risk

### 3.4 Efficacy Outcomes

The analgesic effect of preoperative duloxetine is considered the primary outcome in this study. Postoperative pain was assessed in four studies (Attia and Mansour 2017; Bedin et al. 2017; Altiparmak, Güzel, and Gümüş Demirbilek 2018; Govil et al. 2020), and it was measured by different scales. Attia and Mansour (2017) and Govil et al. (2020) used 11‐point numeric rating scale (NRS), while Bedin et al. (2017) used 11‐point verbal numeric scale (VNS) in which 0 refers to no pain and 10 refers to the worst pain. Altiparmak, Güzel, and Gümüş Demirbilek (2018) used 100 mm visual analogue scale (VAS) to assess the pain intensity.

**FIGURE 4:**
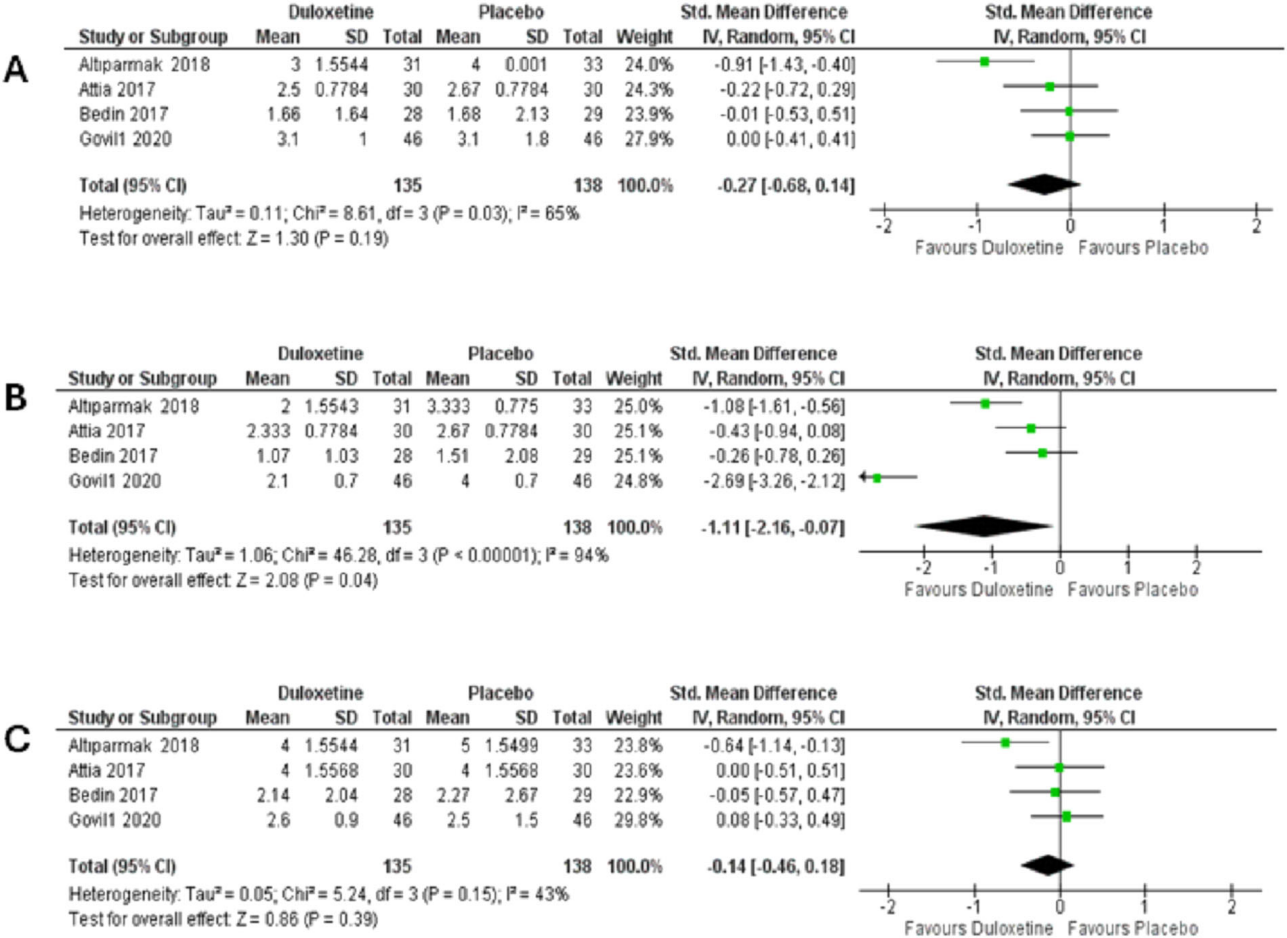
Comparison between duloxetine and placebo in postoperative pain measurements. (A) After 2 h, (B) after 24 h and (C) after 48 h.

Pooled analysis showed that duloxetine significantly reduces pain intensity after 24 h from the operation compared to placebo (SMD = −1.11, 95% CI [−2.16 to −0.07], *p* = 0.04) as shown in Figure 4b. Pooled studies showed marked heterogeneity (*p* < 0.00001, *I* ^2^ = 94%) which could not be resolved neither by subgroup analysis nor sensitivity analysis.

However, the forest plots revealed that duloxetine has no statistically significant effect on reduction of postoperative 2‐ and 48‐h pain severity compared to placebo (SMD = −0.14, 95% CI [−0.46 to 0.18], *p* = 0.39) and (SMD = −0.27, 95% CI [−0.68 to 0.14], *p* = 0.19), respectively shown in Figure 4A,C. Due to marked heterogeneity in both 2 h (*p* = 0.15, *I* ^2^ = 43%) and 24 h (*p* = 0.03, *I* ^2^ = 65%) postoperative pain, we conducted sensitivity analysis to observe that by removing Atiparmak (2018), the heterogeneity was resolved.

**FIGURE 5:**
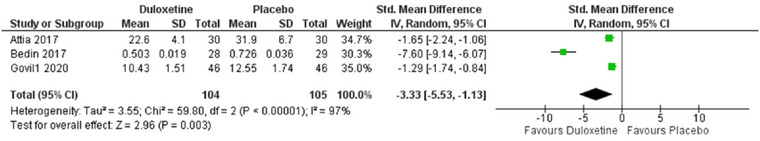
Comparison between duloxetine and placebo in analgesic requirements.

Analgesic requirement or consumption is one of the secondary outcomes that was assessed in three of the included studies (Attia and Mansour 2017; Bedin et al. 2017; Govil et al. 2020). Meta‐analysis revealed that duloxetine shows a significant reduction in the amount of analgesic consumption after 24 h postoperative (MD = −3.33, 95% CI [−5.53 to −1.13], *p* = 0.003). Pooled studies were heterogeneous (*p* < 0.00001, *I* ^2^ = 97%) forest plot shown at Figure 5.

By conducting sensitivity analysis and removing Bedin (2017), heterogeneity was resolved.

**FIGURE 6:**
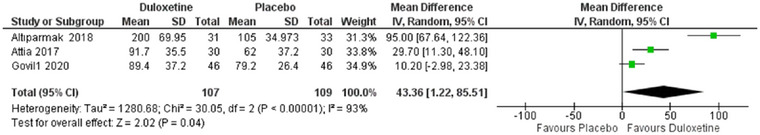
Comparison between duloxetine and placebo in time to first analgesic request.

The other secondary outcome is the time to the first analgesic request. The pooled effect estimate from three studies (Attia and Mansour 2017; Bedin et al. 2017; Govil et al. 2020) favored duloxetine over placebo as the time for the first analgesic need was significantly longer in the duloxetine group (SMD = 43.36, 95% CI [1.22 to 85.51], *p* = 0.04) forest plot shown at Figure 6.

Pooled studies were heterogonous (*p* < 0.00001, *I* ^2^ = 93%) and could not be resolved.

### 3.5 Safety Outcomes

**FIGURE 7:**
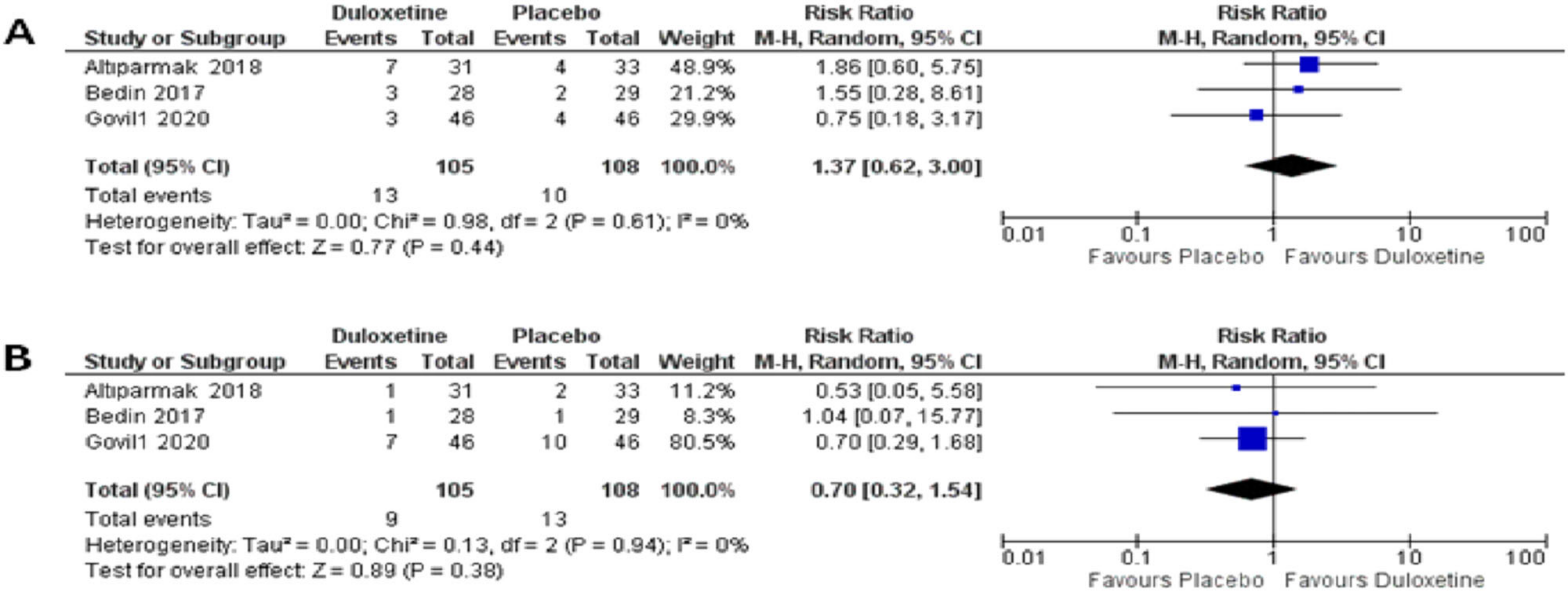
Showing forest plots of pooled analysis regarding nausea and vomiting. (A) Nausea. (B) Vomiting.

Regarding the side effects of duloxetine, nausea and vomiting were evaluated. In three of included studies (Bedin et al. 2017; Altiparmak, Güzel, and Gümüş Demirbilek 2018; Govil et al. 2020), the analysis did not show any statistically significant difference between duloxetine and placebo in patients experiencing nausea or vomiting (RR = 1.37, 95% CI [0.62 to 3.00], *p* = 0.44), and (RR = 0.7, 95% CI [0.32 to 1.54], *p* = 0.38) simultaneously forest plot shown in Figure 7.

Pooled studies were homogenous in both nausea and vomiting outcomes (*p* = 0.61 *I* ^2^ = 0%, *p* = 0.94 *I* ^2^ = 0%), respectively.

**FIGURE 8:**
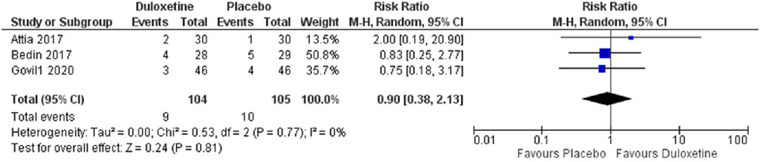
Comparison between duloxetine and placebo regarding risk of somnolence.

The emergence of somnolence was assessed in three of included studies (Attia and Mansour 2017; Bedin et al. 2017; Govil et al. 2020). Results showed that there is no statistical difference among the two groups (RR = 0.9, 95% CI [0.38 to 2.13], *p* = 0.81) and studies were homogenous (*p* = 0.77 *I* ^2^ = 0%) forest plot shown in Figure 8.

**FIGURE 9:**
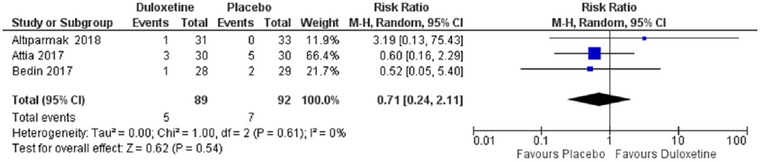
Comparison between duloxetine and placebo regarding risk of itching.

Pooled effect estimate did not favor any group concerning the development of itching (RR = 0.71, 95% CI [0.24 to 2.11], *p* = 0.54) and was homogenous (*p* = 0.61 *I* ^2^ = 0%) forest plot shown in Figure 9.

## 4 Discussion

In this systematic review and meta‐analysis, we tried to present all the currently reported knowledge to evaluate the efficacy and safety of duloxetine, for the reduction of postoperative pain in spine surgeries. We conducted a thorough analysis of the currently available knowledge by reviewing and synthesizing data from seven studies that compared duloxetine with a placebo. Our meta‐analysis results consistently support the positive and well‐tolerated effects of preoperative duloxetine in alleviating pain 48 h after spine surgeries. This is considered the first meta‐analysis that evaluates the effect of duloxetine after spine surgeries specifically.

Patients undergoing spinal surgery are at high risk of acute and persistent postoperative pain. With their preexisting chronic pain and analgesic usage, pain management after spine surgeries become more challenging (Stucky, Gold, and Zhang 2001) sufficient pain management can lead to favorable outcomes such as superior mobility and coordination, quicker recovery, lower risk of complications, and greater patient satisfaction (Lemos et al. 2009; Pugely et al. 2014). Duloxetine is a potent SNRI, the only antidepressant approved for chronic pain (Cipriani et al. 2012). Duloxetine was first noted in 1988 resembling venlafaxine with 100 times potency higher. Sun et al. (2014) found in a rat pain model that duloxetine reduces postoperative pain by elevating spinal levels of norepinephrine and serotonin while activating spinal receptors such as 5‐HT2A or α2‐noradrenergic receptors leading to decreased pain stimulus reaching brain, this may explain rationale behind using Duloxetine in postoperative pain management.

### 4.1 Previous Literature

The majority of published systematic reviews and meta‐analyses evaluated the efficacy of duloxetine after knee or gynecological surgeries (Zhou et al. 2023; Nair et al. 2023; Baradwan et al. 2022; Branton, Hopkins, and Nemec 2021; Yang et al. 2023; Zhang et al. 2023). de Oliveira Filho, Kammer, and Dos Santos (2020) evaluated the effect of duloxetine in treating acute postoperative pain after different surgeries. The results of Filho (2020) showed marked heterogeneity, which was not resolved. On contrary to de Oliveira Filho, Kammer, and Dos Santos (2020) subgroup analysis according to the type of surgery, our results revealed that duloxetine has no significant effect on reducing the pain 24 h postoperative. Time to the first analgesic request outcome was not addressed in de Oliveira Filho, Kammer, and Dos Santos (2020). Schnabel et al. (2021) assessed the efficacy of duloxetine in the treatment of acute and chronic postoperative pain following various surgeries. The results of met analysis were heterogeneous and not explained. Subgroup analysis was not performed, so inference about the effectiveness of duloxetine specifically after spine surgeries cannot be concluded. There are some concerns about analgesic consumption outcome in de Oliveira Filho, Kammer, and Dos Santos (2020) as he extracted the numbers of duloxetine/ etoricoxib group not duloxetine only. The in‐between studies heterogeneity and the variability in the type of surgeries led to marked heterogeneity in the results—which wasn't resolved—and very low quality of evidence in the two previously published SRs (de Oliveira Filho, Kammer, and Dos Santos 2020; Schnabel et al. 2021).

Results of meta‐analysis revealed that preoperative duloxetine has shown a significant reduction in pain severity 48 h after spine surgeries when compared to placebo. However, this reduction appeared to be insignificant 2 and 24 h postoperative. During conducting, the analysis there was a heterogeneity in this outcome which was solved by sensitivity analysis. This could be explained by using the subjective VAS in evaluation of the pain as VAS depends mainly on patients’ cognitive level and their understanding of the questionnaire. Regarding secondary outcomes analysis, reduction of analgesic consumption for the first 24 h after surgery favored duloxetine against placebo. By conducting leave one out analysis and removing Bedin et al. (2017), heterogeneity was resolved. This may be attributed to fentanyl which was administered postoperative. Additionally, duloxetine significantly delayed the time for the first analgesic request for about 45 min.

In terms of safety, our meta‐analysis showed no statistically significant difference between both groups in terms of nausea, vomiting, somnolence and itching. All the results were homogenous.

The two cohort studies evaluate the efficacy of duloxetine on postsurgical chronic pain. Tsuji et al. (2021) study design was a retrospective single cohort that received duloxetine daily for more than 3 months. Results stated that duloxetine was effective in reducing postoperative chronic pain with efficacy rate 100%. Putting into consideration the small sample size and the absence of comparator group, these results is of low evidence and need further investigations. Liu et al. (2023) retrospectively analyzed the records of patients with postoperative axial symptoms taking duloxetine or nondrug therapy for 6 months. Results revealed that duloxetine had significantly better effect on VAS score compared to nondrug therapy. Considering the subjective evaluation of the outcome and the small sample size, the efficacy of duloxetine on postoperative chromic pain needs more future studies with larger sample size.

Two of the included studies showed low risk of bias at all domains (Attia and Mansour 2017; Govil et al. 2020). Altiparmak, Güzel, and Gümüş Demirbilek (2018) had unclear reporting about the concealment of allocation. In Bedin et al. (2017), there was statistically significant difference between groups in perioperative fentanyl consumption which could be a significant covariant. Furthermore, Hiroki et al. (2022) had high risk of bias in the domain of incomplete outcome data as there was unbalanced losses between the two groups: 5/23 (21.7%) in placebo group and 1/23 (4.34%) in duloxetine group. Liu et al. (2023) had some concerns about the assessment of outcome as the adopted method for evaluation was highly subjective.

### 4.2 Limitations

The current study has some limitations. The number of available published RCTs was limited, which made it difficult to conduct subgroup analysis. The in‐between and within study variability led to marked heterogeneity in all efficacy outcomes. Also, pooling of RCTs and cohort studies is a limitation of the current meta‐analysis as cohort studies are a source of bias, although we did this due to the limited published data. Fortunately, the heterogeneity was resolved in analgesic consumption and postoperative pain at 2 and 24 h outcomes by leave one out analysis. The unexplained heterogeneity in the 48‐h postoperative pain and the time needed for the first analgesic use may be due to the different subtypes of spine surgeries, type, dose and time of analgesic used. Finally, we could not assess the publication bias using funnel‐plot‐based methods because they are inaccurate for fewer than 10 studies reporting the same outcome.

## 5 Conclusions

To the best of our knowledge, this systematic review and meta‐analysis represents the first comprehensive investigation into the efficacy and safety of duloxetine for ameliorating pain after spine surgeries. The findings of this study suggest that duloxetine may be effective in reducing pain 24 h after spine surgery. Furthermore, there is a promising effect of duloxetine in treating chronic postoperative pain. However, it is important to acknowledge that further research is warranted to thoroughly evaluate the efficacy and safety of duloxetine for relieving chronic postoperative pain.

## Author Contributions

**Abdulsalam Mohammed Aleid**: conceptualization, investigation, writing–original draft. **Faisal Alshehri**: investigation, validation. **Naif Alasiri**: software, formal analysis. **Fatimah Alhomoud**: data curation, supervision. **Shouq Alsaegh**: software, validation. **Mohammed Alrasheed**: data curation, investigation. **Salem Aljaddua**: investigation, validation. **Ali Alasiri**: visualization, formal analysis. **Asma Boukhari**: methodology, software. **Abdulmonem Ali Alhussain**: formal analysis, software. **Bipin Chaurasia**: writing–review and editing, visualization, validation, supervision. **Saud Nayef Aldanyowi**: data curation, software, methodology.

## Conflicts of Interest

The authors declare no conflicts of interest.

### Peer Review

The peer review history for this article is available at https://publons.com/publon/10.1002/brb3.70217.

## Data Availability

All data are available on the internet.
